# Health-related quality of life in systemic sclerosis compared with other rheumatic diseases: a cross-sectional study

**DOI:** 10.1186/s13075-019-1842-x

**Published:** 2019-02-15

**Authors:** Eun Hye Park, Vibeke Strand, Yoon Jeong Oh, Yeong Wook Song, Eun Bong Lee

**Affiliations:** 1 Division of Rheumatology, Department of Internal Medicine, Seoul National University College of Medicine, Seoul, 03080 Republic of Korea; 2 Division of Immunology/Rheumatology, Stanford University, Palo Alto, CA USA; 3 Department of Internal Medicine, Kangwon National University Hospital, Chuncheon-si, Republic of Korea

**Keywords:** Systemic sclerosis, Quality of life, Disability

## Abstract

**Background:**

Systemic sclerosis (SSc) is a rare autoimmune disease characterized by fibrosis of the skin and the involvement of multiple internal organs. Previous studies reported poorer health-related quality of life (HRQoL) in patients with SSc compared with the general population. However, very little is known about how HRQoL in SSc patients compares with that in patients with other systemic autoimmune diseases, such as rheumatoid arthritis (RA), systemic lupus erythematosus (SLE), and Sjogren’s syndrome (SjS). Thus, the main aim of this study was to compare HRQoL in SSc patients, patients with other rheumatic diseases, and the general population.

**Methods:**

In this cross-sectional study, patients from the rheumatology clinics of Seoul National University Hospital with SSc, RA, SLE, and SjS were enrolled via a random sampling technique. HRQoL was captured by the Short Form (36) health survey (SF-36), the Short Form Six-Dimensional health index (SF-6D), and the EuroQol Five-Dimensional descriptive system (EQ-5D). Demographic characteristics and standardized disease activity for each disease were also obtained. Previously reported data from 600 healthy Koreans were used for the healthy controls. An ANCOVA test was used to compare the SF-36, SF-6D, and EQ-5D values between study subjects with adjustments for age, sex, disease duration, comorbidities, and disease activity status.

**Results:**

One hundred twenty patients were included in each of the SSc, RA, SLE, and SjS cohorts. Patients with rheumatic diseases had significantly lower SF-36, SF-6D, and EQ-5D scores than healthy controls (all *P* < 0.001). After statistical adjustments, SSc patients reported significantly lower mental component summary (MCS) scores than patients with RA (*P* < 0.001) or SLE (*P* = 0.001). Specifically, the mental health and general health domains were significantly lower in SSc patients than reported in RA or SLE patients (*P* < 0.001 and *P* = 0.001, respectively, in both domains). In SSc patients, higher modified Rodnan skin scores (mRSS) correlated with lower MCS scores.

**Conclusions:**

SSc patients report poorer HRQoL than patients with RA or SLE. The extent of skin involvement is associated with poorer HRQoL in SSc patients.

**Trial registration:**

NCT03257878. Registered 22 August 2017

**Electronic supplementary material:**

The online version of this article (10.1186/s13075-019-1842-x) contains supplementary material, which is available to authorized users.

## Background

Systemic sclerosis (SSc) is a chronic autoimmune disorder characterized by autoantibodies, Raynaud’s phenomenon, vascular damage, and fibrosis of the skin and internal organs [1, 2]. Based on the extent of skin involvement, SSc is classified into diffuse cutaneous SSc (dcSSc) and limited cutaneous SSc (lcSSc) [3]. Affected patients experience poor survival owing to derangements in the function of various internal organs, including interstitial lung disease, pulmonary arterial hypertension, renal crisis, gastroesophageal reflux, and congestive heart failure.

Previous studies report poorer health-related quality of life (HRQoL) in SSc patients than in the general population [4–7]. The degree of impairment in SSc patients is similar to or greater than that in those suffering from other chronic conditions such as cardiac and pulmonary disease, hypertension, diabetes mellitus, and depression [5]. However, very little is known about how HRQoL reported by SSc patients compares with that in patients with other systemic rheumatic diseases [6, 8, 9]. The limitations of prior studies include relatively small numbers of patients, those recruited from different disease cohorts, mostly restricted to Caucasian ethnic groups. The objectives of this study were to compare reported HRQoL in patients with SSc, patients with other systemic rheumatic diseases, including rheumatoid arthritis (RA), systemic lupus erythematosus (SLE), and Sjogren’s syndrome (SjS), and healthy subjects, and to assess the clinical factors associated with impaired HRQoL in SSc patients.

## Methods

### Study patients

Patients with SSc, RA, SLE, or SjS (480 patients in total; 120 in each cohort) were randomly selected from the outpatient rheumatology clinics of Seoul National University Hospital between March 2018 and June 2018 via a random number table. All enrolled patients were adults (> 18 years) and fulfilled the standard classification criteria for each disease: the 2013 American College of Rheumatology (ACR)/European League Against Rheumatism (EULAR) criteria for SSc [10], the 1987 ACR criteria [11] and/or the 2010 ACR/EULAR criteria for RA [12], the 1997 ACR criteria [13] and/or the 2012 Systemic Lupus International Collaborating Clinics (SLICC) criteria for SLE [14], and the American–European Consensus Group (AECG) criteria [15] and/or the 2016 ACR/EULAR criteria for SjS [16]. Patients were excluded if they could not give informed consent or were unable to understand or answer the questionnaires. Data on representative Korean healthy controls were obtained from the study of Lee et al., which included 600 healthy Koreans recruited from the general Korean population through a multistage quota sampling method [17]. This study was approved by the institutional review boards and ethics committees at Seoul National University Hospital and conducted in accordance with Good Clinical Practice guidelines and the Declaration of Helsinki. All participants provided written informed consent. The study is listed in ClinicalTrials.gov (NCT03257878).

### Assessment of HRQoL

HRQoL was assessed with the validated Korean version of the Short Form (36) health survey version 2 (SF-36), the Short Form Six-Dimensional health index (SF-6D), and the three-level version of the EuroQol Five-Dimensional descriptive system (EQ-5D-3L). SF-36 is a generic measure of HRQoL and includes eight domains: physical function (PF), role limitations due to physical problems (RP), bodily pain (BP), general health perceptions (GH), vitality (VT), social function (SF), role limitations due to emotional problems (RE), and mental health (MH). These eight domains are summarized as physical component summary (PCS) and mental component summary (MCS) scores with different positive and negative weighting. Each SF-36 domain is scored from 0 to 100, with higher scores representing better health [18]. The psychometric properties of the Korean version of SF-36 in the general population have been demonstrated previously [17, 19], and this validated version was used in this study. EQ-5D-3 L describes general health over five dimensions: mobility, self-care, usual activities, pain/discomfort, and anxiety/depression [20]. Each dimension has three levels: no problems, some or moderate problems, or extreme problems. The EQ-5D descriptive system can be converted into a single summary index by applying a formula that attaches values to each level in each dimension and was calculated using the valuation set from the Korean population [21], with possible EQ-5D scores ranging from − 0.171 to 1.0, where 1.0 represents full health. The EQ visual analogue scale (EQ VAS) records the respondent’s self-reported health on a vertical visual analogue scale (VAS), where 0 and 100 represent the worst and best imaginable health states, respectively. The SF-6D score was calculated from the mean scores across all eight SF-36 domains [22].

Comorbidities and demographic factors, including age, sex, education level, smoking habits, and alcohol consumption, were also collected during the survey. Clinical and laboratory information was obtained through a medical chart review.

### Assessment of disease activity and global disability

RA disease activity was evaluated with a disease activity score that uses erythrocyte sedimentation rate (DAS28-ESR) [23], SLE by the SLE disease activity index 2000 (SLEDAI-2K) [24], and SjS by the EULAR SjS disease activity index (ESSDAI) [25]. In patients with SSc, physical function was measured by the Health Assessment Questionnaire Disability Index (HAQ-DI) [26] and the Scleroderma-Specific HAQ (SHAQ) [27]. The SHAQ combines the disability and pain scales of the HAQ with five scleroderma-specific VASs for digital ulcers, Raynaud’s phenomenon, gastrointestinal (GI) symptoms, lung symptoms, and overall disease severity, with each VAS score scaled from 0 to 3 [28]. The SHAQ is the most widely used and best characterized outcome measure for SSc [28, 29]. A combined SHAQ score was calculated by pooling the eight HAQ-DI domains and the five VASs [30].

Each of the aforementioned disease activity measures produces a single continuous index with defined ranges indicating low, moderate, or high disease activity. For RA patients, low disease activity was defined as DAS28-ESR < 3.2, moderate as DAS28-ESR between 3.2 and 5.1, and high as DAS28-ESR > 5.1 [31]. For SLE patients, SLEDAI-2K < 3, including only one clinical manifestation of rash, alopecia, mucosal ulcers, pleurisy, pericarditis, fever, thrombocytopenia, or leukopenia, was considered indicative of low disease activity; moderate and high disease activity were defined as SLEDAI-2K between 3 and 6, and > 6, respectively [32]. For SjS patients, low, moderate, and high disease activity were defined as ESSDAI < 5, between 5 and 13, and ≥ 14, respectively [33].

For SSc patients, a combined SHAQ score of 0 to 1 indicated mild to moderate disability, a score of 1 to 2 indicated moderate to severe disability, and a score of 2 to 3 indicated severe to very severe disability.

### Statistical analysis

Data are presented as means ± standard deviations (SDs) for continuous variables and as frequencies with percentages for qualitative variables. For continuous variables, the study groups were compared by one-way analysis of variance (ANOVA) with the Scheffe multiple comparisons test. SF-36, SF-6D, and EQ-5D-3 L index scores among the patients with rheumatic diseases and healthy controls were compared by analysis of covariance (ANCOVA), with age and sex adjusted as covariates; for multiple comparisons, corrections were made with the Bonferroni method. ANCOVA was also conducted after full adjustments for age, sex, disease duration, comorbidities, and disease activity states (low, moderate, or high disease activity). Categorical variables were compared with the chi-square or Fisher’s exact tests. Linear regression models were established to assess factors associated with poor HRQoL. Bonferroni corrections were applied across multiple comparisons to prevent α-error accumulation, and *P* < 0.0031 was considered statistically significant. All tests were performed in SPSS version 23 (SPSS Inc., Chicago, IL, USA).

## Results

### Baseline characteristics

Four hudred eighty patients with SSc (*n* = 120), RA (*n* = 120), SLE (*n* = 120), or SjS (*n* = 120) and 600 healthy controls were enrolled in this study. Baseline characteristics differed across the four patient groups and the control group (Table 1). The demographic features of patients with systemic rheumatic diseases were compatible with the known features of each disease group. Table 2 summarizes disease activity indices and disease-specific autoantibodies in patients with SSc, RA, SLE, and SjS. Patients were treated at the discretion of their primary rheumatologist. Overall, RA patients had moderate disease activity with a mean DAS28-ESR score of 3.56 [31]; SLE patients had moderate to high disease activity with a mean SLEDAI-2K score of 6.26 [32] (Table 2). However, mean ESSDAI scores were relatively low (1.52 ± 0.22) because most SjS patients did not have renal or central nervous system involvement (domains highly weighted in the ESSDAI) [33].

**Table 1:** Baseline characteristics of patients with rheumatic diseases and healthy controls

	Group					
	SSc (*n* = 120)	RA (*n* = 120)	SLE (*n* = 120)	SjS (*n* = 120)	Control (*n* = 600)	*P* value*
Age, years, mean (SD)	57.2 (11.2)	58.3 (12.1)	43.0 (14.1)	58.7 (10.9)	44.9 (15.3)	< 0.001
Female, *n* (%)	106 (88.3)	107 (89.2)	107 (89.2)	116 (96.7)	303 (50.5)	< 0.001
Disease duration, years, mean (SD)	11.7 (23.2)	7.6 (6.5)	8.3 (5.6)	6.4 (4.9)	NA	< 0.001
Comorbidities, *n* (%)						
Hypertension	39 (32.5)	38 (31.7)	24 (20.0)	24 (20.0)	99 (16.5)	< 0.001
Diabetes mellitus	12 (10.0)	8 (6.7)	8 (6.7)	7 (5.8)	39 (6.5)	0.713
Dyslipidemia	10 (8.3)	17 (14.2)	19 (15.8)	12 (10.0)	31 (5.2)	< 0.001
Ischemic heart disease	6 (5.0)	3 (2.5)	0 (0)	4 (3.3)	5 (0.8)	0.004
Chronic liver diseases	3 (2.5)	4 (3.3)	7 (5.8)	3 (2.5)	10 (1.7)	0.098
Renal diseases	0 (0)	2 (1.7)	33 (27.5)	4 (3.3)	4 (0.7)	< 0.001
Thyroid diseases	12 (10.0)	8 (6.7)	8 (6.7)	13 (10.8)	6 (1.0)	< 0.001
Education, *n* (%)						< 0.001
University	39 (32.5)	45 (37.5)	69 (57.5)	51 (42.5)	221 (36.8)	
High school	30 (25.0)	45 (37.5)	37 (30.8)	43 (35.8)	278 (46.3)	
Middle school	17 (14.2)	13 (10.8)	7 (5.8)	14 (11.7)	49 (8.2)	
Primary school	21 (17.5)	12 (10.0)	4 (3.3)	11 (9.2)	52 (8.7)	
Uneducated	0 (0)	1 (0.8)	1 (0.8)	1 (0.8)	0 (0)	
BMI, kg/m^2^, mean (SD)	20.6 (4.5)	22.8 (3.0)	22.8 (3.9)	22.6 (5.1)	23.4 (13.2)	0.127
Alcohol, *n* (%)	16 (13.6)	24 (20.0)	32 (26.7)	18 (15.0)		< 0.001
Smoking, *n* (%)	8 (6.8)	11 (9.2)	11 (9.2)	4 (3.3)		0.252
Laboratory findings, mean (SD)						
WBC, mm^3^	7258.6 (202.3)	8079.2 (11,302.2)	5598.1 (2399.8)	5427.7 (1852.2)		0.001
Hemoglobin, g/dL	12.4 (1.4)	12.7 (1.2)	12.4 (1.5)	12.4 (1.2)		0.229
Platelet, mm^3^	240.3 (66.9)	266.2 (64.5)	224.3 (75.2)	217.9 (71.9)		< 0.001
AST, IU/L	23.3 (8.6)	22.6 (9.5)	24.3 (15.2)	24.6 (8.1)		0.473
ALT, IU/L	18.0 (12.3)	20.0 (13.5)	22.6 (24.4)	19.2 (10.9)		0.159
BUN, mg/dL	13.8 (8.2)	14.4 (5.8)	13.7 (6.6)	14.5 (6.3)		0.731
Creatinine, mg/dL	0.9 (1.1)	0.9 (1.6)	0.8 (0.3)	0.9 (1.2)		0.810
ESR, mm/h	30.6 (21.2)	30.6 (20.7)	27.4 (21.5)	27.9 (23.6)		0.520
Hs-CRP, mg/L	0.5 (1.1)	0.6 (1.0)	0.3 (0.5)	0.4 (1.7)		0.378

*SD*, standard deviation; *SSc*, systemic sclerosis; *RA*, rheumatoid arthritis; *SLE*, systemic lupus erythematosus; *SjS*, Sjogren’s syndrome; *BMI*, body mass index; *WBC*, white blood cell; *AST*, aspartate transaminase; *ALT*, alanine transaminase; *BUN*, blood urea nitrogen; *ESR*, erythrocyte sedimentation rate; *hs-CRP*, high sensitivity C-reactive protein

*For continuous variables, statistical significance was tested with one-way analyses of variance and the Scheffe multiple comparison test

**Table 2:** Disease activity indices and disease-specific autoantibodies in patients with rheumatic diseases

Systemic sclerosis	*n* = 120
HAQ-DI, mean (SD)	0.99 (0.85)
SHAQ score, mean (SD)	0.87 (0.68)
Digestive VAS, mean (SD)	0.54 (0.79)
Pulmonary VAS, mean (SD)	0.63 (1.09)
Raynaud’s VAS, mean (SD)	0.78 (0.78)
Digital ulcer VAS, mean (SD)	0.41 (0.71)
Overall disease severity VAS, mean (SD)	1.05 (0.88)
ANA, *n* (%)	105 (87.5)
Anti-topoisomerase I antibody, *n* (%)	49 (40.8)
Anti-centromere antibody, *n* (%)	23 (19.2)
Rheumatoid arthritis	*n* = 120
DAS28 (ESR), mean (SD)	3.56 (0.12)
Patient global health, mean (SD)	35.21 (2.17)
Rheumatoid factor, *n* (%)	109 (90.8)
Anti-citrullinated protein antibody, *n* (%)	76 (63.3)
Systemic lupus erythematosus	*n* = 120
SLEDAI-2K, mean (SD)	6.26 (1.22)
ANA, *n* (%)	116 (96.7)
Anti-Smith antibody, *n* (%)	47 (39.2)
Anti-ds DNA antibody, *n* (%)	90 (75.0)
C3, mg/dL, mean (SD)	74.22 (4.15)
C4, mg/dL, mean (SD)	12.78 (1.15)
Sjogren’s syndrome	*n* = 120
ESSDAI, mean (SD)	1.52 (0.22)
ANA, *n* (%)	110 (91.7)
Anti-SSA/Ro autoantibody, *n* (%)	69 (57.5)
Anti-SSB/La autoantibody, *n* (%)	81 (67.5)

*HAQ-DI*, Health Assessment Questionnaire Disability Index; *SHAQ*, Scleroderma-Specific Health Assessment Questionnaire; *ANA*, antinuclear antibodies; *DAS28-ESR*, disease activity score evaluated with erythrocyte sedimentation rate; *SLEDAI-2K*, systemic lupus erythematosus disease activity index 2000; *ESSDAI*, EULAR Sjogren’s syndrome disease activity index

SSc patients reported a mean HAQ-DI score of 0.99 and a mean combined SHAQ score of 0.87. Among SSc patients, 79 (65.8%) had dcSSc and the remaining had lcSSc, with mean modified Rodnan skin scores (mRSS) of 15.6 (Additional file 1: Table S1). Raynaud’s phenomenon was the most common clinical manifestation at the time of assessment (91.7%). Regarding GI symptoms, 60 patients (50.0%) had reflux symptoms and 38 (31.7%) dysphagia. The proportions of SSc patients with interstitial lung disease or pulmonary arterial hypertension, the two leading causes of morbidity and mortality in SSc [34], were 57.5% and 10.8%, respectively.

### HRQoL in patients with systemic rheumatic diseases and in healthy controls

**Fig. 1:**
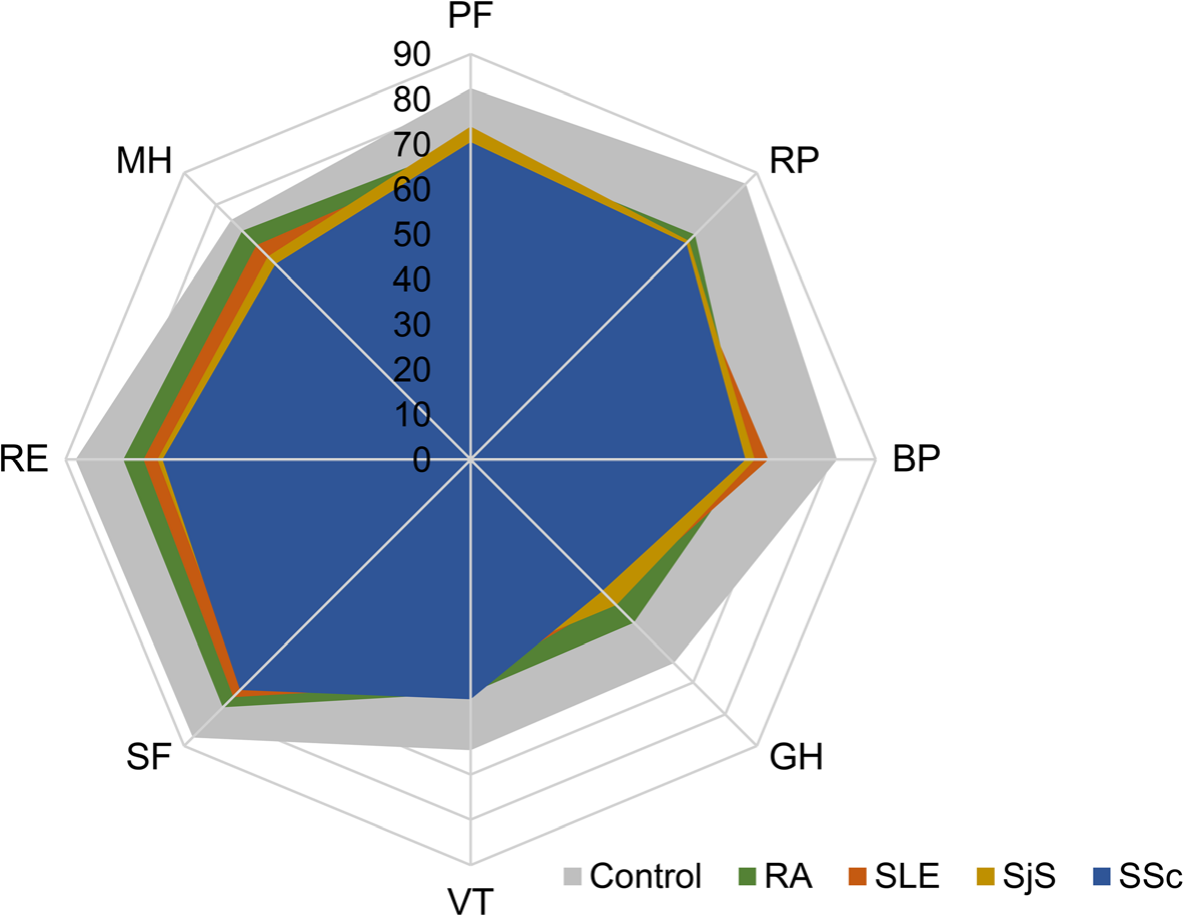
Comparison of the SF-36 subscales adjusted by age and sex. SF-36, Short Form (36) health survey; SSc, systemic sclerosis; RA, rheumatoid arthritis; SLE, systemic lupus erythematosus; SjS, Sjogren’s syndrome; PF, physical function; RP, role–physical; BP, bodily pain; GH, general health perception; VT, vitality; SF, social function; RE, role–emotional; MH, mental health

Patients with rheumatic diseases reported significantly lower SF-36 scores across all domains (*P* < 0.001 in all domains) (Fig. 1), lower SF-6D scores (*P* < 0.001), and lower EQ-5D-3 L scores (*P* < 0.001) than healthy controls after adjustments for age and sex (Table 3). Patients with SSc had significantly lower SF-36 MCS scores than patients with RA (age- and sex-adjusted scores, 43.0 ± 0.9 vs. 48.9 ± 0.9; *P* < 0.001). Specifically, MH domain scores were significantly lower in SSc than in RA patients (age- and sex-adjusted scores, 61.3 ± 1.8 vs. 71.7 ± 1.8, *P* < 0.001) (Fig. 1) (Table 3). Among the physical domain scores, SSc patients reported lower scores in the GH domain than RA patients (age- and sex-adjusted scores, 41.4 ± 1.8 vs. 51.3 ± 1.8, *P* < 0.001). Further ANCOVA analyses with additional adjustments for disease duration, comorbidities, and disease activity (low, moderate, or high disease activity level) yielded results similar to those of the main analysis (Table 4). SSc patients demonstrated significantly lower scores in GH (fully adjusted scores, 37.1 ± 1.8 vs. 48.1 ± 1.8, *P* < 0.001) and MH (fully adjusted scores, 58.3 ± 2.0 vs. 69.8 ± 2.0, *P* < 0.001) domains than RA patients; BP (56.3 ± 2.3 vs. 69.6 ± 2.6, *P* < 0.001), GH (37.1 ± 1.8 vs. 46.8 ± 2.1, *P* = 0.001), and MH (58.3 ± 2.0 vs. 68.6 ± 2.3, *P* = 0.001) domains were also significantly lower in SSc patients than in SLE patients. A sensitivity analysis also revealed lower MCS scores in SSc patients than in RA (*P* < 0.001) or SLE (*P* = 0.001) patients. SSc patients reported markedly lower EQ-5D-3 L scores than RA and SLE patients (*P* < 0.001). SSc and SjS patients had similar trends in SF-36 domains; SjS patients demonstrated significantly lower scores in the VT (fully adjusted scores, 38.3 ± 2.1 vs. 48.7 ± 2.0, *P* < 0.001) domain than SSc patients.

**Table 3:** Subscales of the SF-36, SF-6D, and EQ-5D-3 L scores adjusted by age and sex

	Group						Post hoc *P* value			
	SSc (*n* = 120)	RA (*n* = 120)	SLE (*n* = 120)	SjS (*n* = 120)	Control (*n* = 600)	*P*	SSc vs. RA	SSc vs. SLE	SSc vs. SjS	SSc vs. control
PF	70.5 (2.2)	70.3 (2.2)	68.2 (2.2)	73.9 (2.2)	82.4 (1.0)	< 0.001	0.952	0.464	0.265	*< 0.001*
RP	67.8 (2.3)	70.5 (2.3)	65.8 (2.3)	68.6 (2.4)	86.5 (1.1)	< 0.001	0.393	0.541	0.801	*< 0.001*
BP	61.1 (2.2)	61.4 (2.2)	66.1 (2.2)	63.1 (2.3)	81.4 (1.0)	< 0.001	0.920	0.114	0.499	*< 0.001*
GH	41.4 (1.8)	51.3 (1.8)	44.2 (1.8)	45.8 (1.8)	63.9 (0.8)	< 0.001	*< 0.001*	0.261	0.074	*< 0.001*
VT	53.2 (1.9)	51.8 (1.9)	49.7 (1.9)	45.8 (1.9)	64.6 (0.9)	< 0.001	0.610	0.196	0.005	*< 0.001*
SF	72.4 (2.1)	77.8 (2.1)	74.6 (2.1)	71.3 (2.1)	87.3 (1.0)	< 0.001	0.058	0.440	0.703	*< 0.001*
RE	68.4 (2.3)	77.2 (2.3)	72.7 (2.3)	69.6 (2.4)	87.7 (1.1)	< 0.001	0.006	0.199	0.713	*< 0.001*
MH	61.3 (1.8)	71.7 (1.8)	67.2 (1.8)	63.7 (1.8)	75.2 (0.8)	< 0.001	*< 0.001*	0.020	0.326	*< 0.001*
PCS	44.6 (0.8)	45.3 (0.8)	44.8 (0.8)	46.9 (0.8)	51.0 (0.4)	< 0.001	0.511	0.827	0.025	*< 0.001*
MCS	43.0 (0.9)	48.9 (0.9)	46.6 (0.9)	44.2 (0.9)	50.8 (0.4)	< 0.001	*< 0.001*	0.006	0.316	*< 0.001*
SF-6D	0.70 (0.01)	0.73 (0.01)	0.72 (0.01)	0.71 (0.01)	0.79 (0.00)	< 0.001	0.056	0.310	0.669	*< 0.001*
EQ-5D-3 L	63.98 (1.55)	69.95 (1.56)	67.49 (1.55)	64.84 (1.58)	77.88 (0.72)	< 0.001	0.005	0.110	0.684	*< 0.001*

*SSc*, systemic sclerosis; *RA*, rheumatoid arthritis; *SLE*, systemic lupus erythematosus; *SjS*, Sjogren’s syndrome; *PF*, physical function; *RP*, role–physical; *BP*, bodily pain; *GH*, general health perception; *VT*, vitality; *SF*, social function; *RE*, role–emotional; *MH*, mental health; *PCS*, physical component score; *MCS*, mental component score; *SF-36*, Short Form (36) health survey; *SF-6D*, Short Form Six-Dimensional health index; *EQ-5D-3L*, three-level version of the EuroQol Five-Dimensional descriptive system

Statistically significant results after the Bonferroni corrections are printed in italics

**Table 4:** Subscales of SF-36, SF-6D, and EQ-5D-3 L scores adjusted by age, sex, disease duration, comorbidities, and disease activity state

	Group					Post hoc *P* value		
	SSc (*n* = 120)	RA (*n* = 120)	SLE (*n* = 120)	SjS (*n* = 120)	*P*	SSc vs. RA	SSc vs. SLE	SSc vs. SjS
Physical function	66.2 (2.3)	67.6 (2.3)	67.3 (2.6)	61.3 (2.4)	0.245	0.682	0.773	0.136
Role–physical	64.7 (2.5)	70.3 (2.6)	66.7 (2.9)	58.0 (2.7)	0.011	0.123	0.621	0.062
Bodily pain	56.3 (2.3)	59.7 (2.3)	69.6 (2.6)	51.9 (2.4)	< 0.001	0.302	*< 0.001*	0.185
General health	37.1 (1.8)	48.1 (1.8)	46.8 (2.1)	38.3 (1.9)	< 0.001	*< 0.001*	*0.001*	0.632
Vitality	48.7 (2.0)	48.8 (2.1)	50.4 (2.3)	38.3 (2.1)	< 0.001	0.960	0.592	*< 0.001*
Social function	70.1 (2.5)	77.1 (2.5)	76.0 (2.8)	64.2 (2.6)	0.002	0.042	0.128	0.095
Role–emotional	66.4 (2.7)	76.8 (2.7)	74.4 (3.0)	60.7 (2.8)	< 0.001	0.005	0.057	0.129
Mental health	58.3 (2.0)	69.8 (2.0)	68.6 (2.3)	58.2 (2.1)	< 0.001	*< 0.001*	*0.001*	0.947
Physical component score	42.8 (0.8)	44.3 (0.8)	45.3 (0.9)	42.3 (0.8)	0.068	0.164	0.042	0.675
Mental component score	41.8 (1.1)	48.3 (1.1)	47.4 (1.2)	41.5 (1.1)	< 0.001	*< 0.001*	*0.001*	0.846
SF-6D	0.68 (0.01)	0.72 (0.01)	0.72 (0.01)	0.66 (0.01)	< 0.001	0.015	0.010	0.082
EQ-5D-3 L	0.74 (0.02)	0.81 (0.02)	0.83 (0.02)	0.76 (0.02)	< 0.001	*< 0.001*	*< 0.001*	0.212

*SSc*, systemic sclerosis; *RA*, rheumatoid arthritis; *SLE*, systemic lupus erythematosus; *SjS*, Sjogren’s syndrome; *SF-36*, Short Form (36) health survey; *SF-6D*, Short Form Six-Dimensional health index; *EQ-5D-3L*, three-level version of the EuroQol Five-Dimensional descriptive system

Statistically significant results after the Bonferroni corrections are printed in italics

Post hoc *P* values for comparisons of SF-36 domains, SF-6D, and EQ-5D-3 L scores among patients with rheumatic diseases other than SSc are demonstrated in Additional file 2: Table S2 and Additional file 3: Table S3.

### Factors associated with HRQoL

Table 5 shows the results of a linear regression on factors associated with poorer HRQoL in SSc patients. Body mass index (BMI) was positively correlated with SF-36 PCS scores in SSc patients (beta = 0.32, *P* = 0.022), whereas disease duration (beta = − 0.08, *P* = 0.009) and SHAQ digestive (beta = − 3.69, *P* < 0.001), pulmonary (beta = − 2.68, *P* = 0.004), and disease severity (beta = − 3.18, *P* = 0.003) VASs were negatively correlated with SF-36 PCS scores in SSc patients. mRSS was significantly associated with both PCS (beta = − 0.25, *P* = 0.001) and MCS (beta = − 0.28, *P* = 0.021) scores in SSc patients. EQ-5D-3 L scores were also significantly associated with mRSS (beta = − 0.005, *P* = 0.021) and the SHAQ disease severity VAS (beta = − 0.098, *P* = 0.003).

**Table 5:** Linear regression analyses of factors associated with SF-36 and EQ-5D-3 L scores in patients with systemic sclerosis

	SF-36						EQ-5D-3 L		
	Physical component score			Mental component score					
	Slope (SE)	Beta	*P*	Slope (SE)	Beta	*P*	Slope (SE)	Beta	*P*
Age	− 0.06 (0.06)	− 0.05	0.352	0.05 (0.10)	0.04	0.629	− 0.001 (0.002)	− 0.06	0.430
Sex	0.44 (2.02)	0.01	0.829	1.83 (3.33)	0.05	0.583	0.039 (0.062)	0.04	0.534
BMI	0.32 (0.14)	0.13	0.022	− 0.17 (0.22)	− 0.07	0.448	0.005 (0.004)	0.10	0.199
Disease duration	− 0.08 (0.03)	− 0.15	0.009	− 0.03 (0.05)	− 0.06	0.516	− 0.002 (0.001)	− 0.13	0.072
Subset (lcSSc)	4.13 (1.39)	0.16	0.004	4.34 (2.29)	0.17	0.061	0.090 (0.043)	0.15	0.039
mRSS	− 0.25 (0.07)	− 0.21	0.001	− 0.28 (0.12)	− 0.24	0.021	− 0.005 (0.002)	− 0.19	0.021
Raynaud’s VAS	− 1.15 (0.95)	− 0.08	0.229	− 2.40 (1.57)	− 0.16	0.129	− 0.016 (0.029)	0.05	0.579
Digestive VAS	− 3.69 (1.02)	− 0.24	< 0.001	0.11 (1.69)	0.01	0.947	− 0.051 (0.031)	− 0.14	0.105
Pulmonary VAS	− 2.68 (0.90)	− 0.19	0.004	− 0.04 (1.48)	− 0.01	0.979	− 0.051 (0.028)	− 0.15	0.066
Digital ulcer VAS	− 0.68 (0.91)	− 0.04	0.455	3.23 (1.50)	0.20	0.084	0.015 (0.028)	0.04	0.581
Disease severity VAS	− 3.18 (1.04)	− 0.22	0.003	− 3.18 (1.70)	− 0.23	0.064	− 0.098 (0.032)	− 0.30	0.003

*SF-36*, Short Form (36) health survey; *EQ-5D-3L*, three-level version of the EuroQol Five-Dimensional descriptive system; *BMI*, body mass index; *lcSSc*, limited cutaneous systemic sclerosis; *VAS*, visual analogue scale; *PAH*, pulmonary arterial hypertension; *ILD*, interstitial lung disease

## Discussion

In this study, we evaluated HRQoL reported by SSc patients compared with that reported by patients with RA, SLE, and SjS, and healthy controls. This is the first study investigating comparative HRQoL in patients with autoimmune diseases from an Asian population. Patients with SSc had poorer HRQoL after adjustments for age, sex, disease duration, comorbidities, and disease activity state. Specifically, SSc patients reported greater impairments in mental health and poorer perception of general health than RA and SLE patients. The extent of skin involvement in SSc patients was associated with reduced physical and mental HRQoL scores.

Overall, SSc patients reported a significantly poorer HRQoL than healthy controls. This result is consistent with that of a previous systematic review that indicated that HRQoL was significantly impaired in SSc patients, with pooled SF-36 PCS scores more than one SD below the general population norm (38.3; 95% CI, 35.2–41.5) and pooled MCS scores approximately half a SD below the general population norm (46.6; 95% CI, 44.2–49.1) [4]. In this study, SSc patients reported poorer MH scores than RA or SLE patients. This may partly be explained by the different clinical features and the different patterns of the disease course across diseases. In contrast to RA patients who usually present with single organ involvement—the joints [35], patients with SSc and SLE have multiple organ involvements [3, 36], which may significantly affect HRQoL. Furthermore, SLE is characterized by intermittent flares [36] whereas SSc has a more progressive disease course with some experiencing rapid progression and others a more indolent course. SSc patients are known to suffer from psychological stress owing to cosmetic disfigurements, including tight and shiny skin, a beaked nose, facial telangiectasias, and loss of the vermillion border of the lips [37]. In a cross-sectional study of 127 US women with SSc, the degree of dissatisfaction with body image was even greater than in those with severe burn injuries [38]. Depression, anxiety, poor self-image, sexual dysfunction, and fear of disease progression were also reported to be prevalent in SSc patients [38–40]. Impaired mental health and poor perception of general health appear to be related to the psychological stress these patients experience.

In this study, SSc and SjS patients showed similar trends in reported HRQoL except lower VT scores in SjS. SjS patients are known to have a poor HRQoL with anxiety and depression [41, 42]. Pain and fatigue are known as primary factors for lower HRQoL in SjS patients [42]. However, special care should be taken in the interpretation of these SjS data, because our patient population was composed of those with low ESSDAI scores, whereas patients with SSc, SLE, and RA predominantly showed moderate to high disease activity.

There have been few comparative studies of HRQoL between patients with SSc and patients with other systemic rheumatic diseases [6, 8, 9]. In a cross-sectional study of incident patients from four different cohorts, Greenfield et al. reported that inflammatory myopathy patients have the worst physical and mental HRQoL at disease onset, SSc and RA patients have considerably impaired physical but less so mental HRQoL, and SLE patients have moderate impairments in both physical and mental HRQoL [9]. However, this study included only patients in the early stages of disease, whereas comorbidities that develop over the long-term course of the disease seriously affect HRQoL, as illustrated by the present study. Johnson et al. reported that HRQoL was similar across groups of rheumatology patients, including those with SSc, RA, SLE, and psoriatic arthritis (PsA) [8]. However, SSc patients with joint involvement reported significantly higher global disability than patients with PsA and experienced more severe pain than patients with RA. In the present study, 38.3% of SSc patients had arthritis (Additional file 1: Table S1) and experienced more pain in general than SLE patients. Danieli et al. reported that perceptions of HRQoL were not statistically different between patients with RA and SSc, but that dcSSc patients had significantly worse scores in the GH and MH domains than patients with RA [6]. Interestingly, these results are partly in line with our current finding that SSc patients specifically have worse perceptions of their general and mental health than those with RA and SLE.

Our results demonstrate that the degree of skin involvement (mRSS) has a negative impact on both PCS and MCS scores in SSc patients. Impaired physical HRQoL was associated with longer disease duration, belonging to the dcSSc patient subset, and with GI and pulmonary involvement, in addition to mRSS. These results are consistent with previously reported results. The extent of skin involvement, tendon/joint contracture, and damage to the heart and peripheral vascular systems are associated with poorer functioning and HRQoL in patients with SSc [7]. Digital ulcers have a negative impact on the MCS score in SSc patients [43], whereas pulmonary fibrosis negatively correlates with the PCS score [44]. Involvement of the GI tract in SSc patients is associated with lower HRQoL, disability, and depressed mood [8, 45, 46]. Comparisons between SSc disease subtypes have indicated that dcSSc patients had greater functional impairments, with lower PCS scores, than those with lcSSc [47]. These results suggest that the extent of skin thickening and disease duration, which influences the involvement of the internal organs, impact the PCS scores. Recently, a large prospective cohort study from the DeSSipher project within the EUSTAR group, which included 944 SSc patients with SHAQ scores, demonstrated that SSc patients perceive dyspnea, pain, digital ulcers, muscle weakness, and GI symptoms as the main factors driving their level of disability [48].

The results of this study indicate that we should pay more attention to the reported HRQoL and psychological status in patients with SSc. Psychological support and education about the disease may help improve SSc patients’ HRQoL. Furthermore, early diagnosis and assessment of organ involvements, as well as early, tailored, appropriate organ-based treatment would be advantageous to ensure better quality of life in SSc patients.

There are several limitations to this study. First, the demographic features differed among the disease groups. For example, as expected, SLE patients were younger than patients in the other disease groups. To overcome this limitation, we controlled for major demographic factors during the statistical analysis. Second, the questionnaires used in this study (SF-36, SF-6D, and EQ-5D-3 L) may not be able to assess specific impairments related to particular types of rheumatologic patients. However, these generic tools have been used previously to assess HRQOL in these different diseases, and allow comparisons between them and the general population. Third, the study populations were composed of Korean patients only. Therefore, the generalizability of our results to other ethnic groups needs to be confirmed. Fourth, since this is a cross-sectional study, we could not evaluate the effect of the immunosuppressive agents on HRQoL outcomes. Future studies are needed to evaluate the impact of the immunosuppressive treatment on HRQoL in patients with rheumatologic diseases.

## Conclusions

In summary, SSc patients have poorer HRQoL than both healthy controls and patients with RA or SLE, and specifically, worse perceptions of their general and mental health. The extent of skin involvement is an important factor associated with reduced physical and mental HRQoL in SSc patients; lower BMI, prolonged disease duration, having dcSSc, and the presence of gastrointestinal and pulmonary involvement are associated with poorer PCS scores.

## Additional file

Additional file 1: **Table S1.** Clinical features of patients with SSc (n=120). (DOCX 16 kb)

Additional file 2: **Table S2.** SF-36 domains, SF-6D, and EQ-5D-3L scores adjusted by age and sex. (DOCX 19 kb)

Additional file 3: **Table S3.** SF-36 domains, SF-6D, and EQ-5D-3L scores adjusted by age, sex, disease duration, comorbidities, and disease activity states. (DOCX 18 kb)

## Abbreviations

ACR: American College of Rheumatology
AECG: American–European Consensus Group
ANCOVA: Analysis of covariance
ANOVA: One-way analysis of variance
BP: Bodily pain
DAS28-ESR: Disease activity score using erythrocyte sedimentation rate
EQ VAS: EQ visual analogue scale
EQ-5D: EuroQol Five-Dimensional descriptive system
EQ-5D-3L: Three-level version of EQ-5D
ESSDAI: EULAR Sjogren’s syndrome disease activity index
EULAR: European League Against Rheumatism
GH: General health perceptions
GI: Gastrointestinal
HAQ-DI: Health Assessment Questionnaire Disability Index
HRQoL: Health-related quality of life
MCS: Mental component summary scores
MH: Mental health
mRSS: Modified Rodnan skin scores
PCS: Physical component summary scores
PF: Physical function
PsA: Psoriatic arthritis
RA: Rheumatoid arthritis
RE: Role limitations due to emotional problems
RP: Role limitations due to physical problems
SF: Social function
SF-36: Short Form (36) health survey
SF-6D: Short Form Six-Dimensional health index
SHAQ: Scleroderma-Specific HAQ
SLE: Systemic lupus erythematosus
SLEDAI-2K: SLE disease activity index 2000
SLICC: Systemic Lupus International Collaborating Clinics
SjS: Sjogren’s syndrome
SSc: Systemic sclerosis
VAS: Visual analogue scale
VT: Vitality

## References

[1] Varga J, Trojanowska M, Kuwana M (2017). Pathogenesis of systemic sclerosis: recent insights of molecular and cellular mechanisms and therapeutic opportunities. J Scleroderma Relat Disord.

[2] Hudson M, Fritzler MJ, Baron M, Canadian Scleroderma Research G (2010). Systemic sclerosis: establishing diagnostic criteria. Medicine (Baltimore).

[3] LeRoy EC, Black C, Fleischmajer R, Jablonska S, Krieg T, Medsger TA, Rowell N, Wollheim F (1988). Scleroderma (systemic sclerosis): classification, subsets and pathogenesis. J Rheumatol.

[4] Hudson M, Thombs BD, Steele R, Panopalis P, Newton E, Baron M, Canadian Scleroderma Research G (2009). Health-related quality of life in systemic sclerosis: a systematic review. Arthritis Rheum.

[5] Hudson M, Thombs BD, Steele R, Panopalis P, Newton E, Baron M, Canadian Scleroderma Research G (2009). Quality of life in patients with systemic sclerosis compared to the general population and patients with other chronic conditions. J Rheumatol.

[6] Danieli E, Airo P, Bettoni L, Cinquini M, Antonioli CM, Cavazzana I, Franceschini F, Cattaneo R (2005). Health-related quality of life measured by the Short Form 36 (SF-36) in systemic sclerosis: correlations with indexes of disease activity and severity, disability, and depressive symptoms. Clin Rheumatol.

[7] Chan PT, Mok CC, Chan KL, Ho LY (2014). Functioning and health-related quality of life in Chinese patients with systemic sclerosis: a case-control study. Clin Rheumatol.

[8] Johnson SR, Glaman DD, Schentag CT, Lee P (2006). Quality of life and functional status in systemic sclerosis compared to other rheumatic diseases. J Rheumatol.

[9] Greenfield J, Hudson M, Vinet E, Fortin PR, Bykerk V, Pineau CA, Wang M, Bernatsky S, Baron M, Canadian Scleroderma Research G (2017). A comparison of health-related quality of life (HRQoL) across four systemic autoimmune rheumatic diseases (SARDs). PLoS One.

[10] van den Hoogen F, Khanna D, Fransen J, Johnson SR, Baron M, Tyndall A, Matucci-Cerinic M, Naden RP, Medsger TA, Carreira PE (2013). 2013 classification criteria for systemic sclerosis: an American College of Rheumatology/European League against Rheumatism collaborative initiative. Ann Rheum Dis.

[11] Arnett FC, Edworthy SM, Bloch DA, McShane DJ, Fries JF, Cooper NS, Healey LA, Kaplan SR, Liang MH, Luthra HS (1988). The American Rheumatism Association 1987 revised criteria for the classification of rheumatoid arthritis. Arthritis Rheum.

[12] Aletaha D, Neogi T, Silman AJ, Funovits J, Felson DT, Bingham CO, Birnbaum NS, Burmester GR, Bykerk VP, Cohen MD (2010). 2010 rheumatoid arthritis classification criteria: an American College of Rheumatology/European League Against Rheumatism collaborative initiative. Arthritis Rheum.

[13] Hochberg MC (1997). Updating the American College of Rheumatology revised criteria for the classification of systemic lupus erythematosus. Arthritis Rheum.

[14] Petri M, Orbai AM, Alarcon GS, Gordon C, Merrill JT, Fortin PR, Bruce IN, Isenberg D, Wallace DJ, Nived O (2012). Derivation and validation of the Systemic Lupus International Collaborating Clinics classification criteria for systemic lupus erythematosus. Arthritis Rheum.

[15] Vitali C, Bombardieri S, Jonsson R, Moutsopoulos HM, Alexander EL, Carsons SE, Daniels TE, Fox PC, Fox RI, Kassan SS (2002). Classification criteria for Sjogren’s syndrome: a revised version of the European criteria proposed by the American-European Consensus Group. Ann Rheum Dis.

[16] Shiboski CH, Shiboski SC, Seror R, Criswell LA, Labetoulle M, Lietman TM, Rasmussen A, Scofield H, Vitali C, Bowman SJ (2017). 2016 American College of Rheumatology/European League Against Rheumatism classification criteria for primary Sjogren's syndrome: a consensus and data-driven methodology involving three international patient cohorts. Ann Rheum Dis.

[17] Kim SH, Jo MW, Lee SI (2013). Psychometric properties of the Korean short form-36 health survey version 2 for assessing the general population. Asian Nurs Res (Korean Soc Nurs Sci).

[18] Ware JE, Sherbourne CD (1992). The MOS 36-item short-form health survey (SF-36). I. Conceptual framework and item selection. Med Care.

[19] Han CW, Lee EJ, Iwaya T, Kataoka H, Kohzuki M (2004). Development of the Korean version of Short-Form 36-Item Health Survey: health related QOL of healthy elderly people and elderly patients in Korea. Tohoku J Exp Med.

[20] Rabin R, de Charro F (2001). EQ-5D: a measure of health status from the EuroQol Group. Ann Med.

[21] Lee YK, Nam HS, Chuang LH, Kim KY, Yang HK, Kwon IS, Kind P, Kweon SS, Kim YT (2009). South Korean time trade-off values for EQ-5D health states: modeling with observed values for 101 health states. Value Health.

[22] Ara R, Brazier J (2009). Predicting the short form-6D preference-based index using the eight mean short form-36 health dimension scores: estimating preference-based health-related utilities when patient level data are not available. Value Health.

[23] van der Heijde DM, van ’t Hof M, van Riel PL, van de Putte LB (1993). Development of a disease activity score based on judgment in clinical practice by rheumatologists. J Rheumatol.

[24] Gladman DD, Ibanez D, Urowitz MB (2002). Systemic lupus erythematosus disease activity index 2000. J Rheumatol.

[25] Seror R, Ravaud P, Bowman SJ, Baron G, Tzioufas A, Theander E, Gottenberg JE, Bootsma H, Mariette X, Vitali C (2010). EULAR Sjogren’s syndrome disease activity index: development of a consensus systemic disease activity index for primary Sjogren’s syndrome. Ann Rheum Dis.

[26] Merkel PA, Herlyn K, Martin RW, Anderson JJ, Mayes MD, Bell P, Korn JH, Simms RW, Csuka ME, Medsger TA (2002). Measuring disease activity and functional status in patients with scleroderma and Raynaud’s phenomenon. Arthritis Rheum.

[27] Steen VD, Medsger TA (1997). The value of the Health Assessment Questionnaire and special patient-generated scales to demonstrate change in systemic sclerosis patients over time. Arthritis Rheum.

[28] Johnson SR, Hawker GA, Davis AM (2005). The health assessment questionnaire disability index and scleroderma health assessment questionnaire in scleroderma trials: an evaluation of their measurement properties. Arthritis Rheum.

[29] Pope J (2011). Measures of systemic sclerosis (scleroderma): Health Assessment Questionnaire (HAQ) and Scleroderma HAQ (SHAQ), physician- and patient-rated global assessments, Symptom Burden Index (SBI), University of California, Los Angeles, Scleroderma Clinical Trials Consortium Gastrointestinal Scale (UCLA SCTC GIT) 2.0, Baseline Dyspnea Index (BDI) and Transition Dyspnea Index (TDI) (Mahler’s Index), Cambridge Pulmonary Hypertension Outcome Review (CAMPHOR), and Raynaud’s Condition Score (RCS). Arthritis Care Res (Hoboken).

[30] Georges C, Chassany O, Mouthon L, Tiev K, Toledano C, Meyer O, Marjanovic Z, Heneggar C, Papo T, Crickx B (2005). Validation of French version of the Scleroderma Health Assessment Questionnaire (SSc HAQ). Clin Rheumatol.

[31] Anderson J, Caplan L, Yazdany J, Robbins ML, Neogi T, Michaud K, Saag KG, O'Dell JR, Kazi S (2012). Rheumatoid arthritis disease activity measures: American College of Rheumatology recommendations for use in clinical practice. Arthritis Care Res (Hoboken).

[32] Polachek A, Gladman DD, Su J, Urowitz MB (2017). Defining low disease activity in systemic lupus erythematosus. Arthritis Care Res (Hoboken).

[33] Seror R, Bootsma H, Saraux A, Bowman SJ, Theander E, Brun JG, Baron G, Le Guern V, Devauchelle-Pensec V, Ramos-Casals M (2016). Defining disease activity states and clinically meaningful improvement in primary Sjogren’s syndrome with EULAR primary Sjogren’s syndrome disease activity (ESSDAI) and patient-reported indexes (ESSPRI). Ann Rheum Dis.

[34] Tyndall AJ, Bannert B, Vonk M, Airo P, Cozzi F, Carreira PE, Bancel DF, Allanore Y, Muller-Ladner U, Distler O (2010). Causes and risk factors for death in systemic sclerosis: a study from the EULAR Scleroderma Trials and Research (EUSTAR) database. Ann Rheum Dis.

[35] Lee DM, Weinblatt ME (2001). Rheumatoid arthritis. Lancet.

[36] La Paglia GMC, Leone MC, Lepri G, Vagelli R, Valentini E, Alunno A, Tani C (2017). One year in review 2017: systemic lupus erythematosus. Clin Exp Rheumatol.

[37] Amin K, Clarke A, Sivakumar B, Puri A, Fox Z, Brough V, Denton CP, Peter EM, Butler P (2011). The psychological impact of facial changes in scleroderma. Psychol Health Med.

[38] Benrud-Larson LM, Heinberg LJ, Boling C, Reed J, White B, Wigley FM, Haythornthwaite JA (2003). Body image dissatisfaction among women with scleroderma: extent and relationship to psychosocial function. Health Psychol.

[39] Faezi ST, Paragomi P, Shahali A, Akhlaghkhah M, Akbarian M, Akhlaghi M, Kheirandish M, Gharibdoost F (2017). Prevalence and severity of depression and anxiety in patients with systemic sclerosis: an epidemiologic survey and investigation of clinical correlates. J Clin Rheumatol.

[40] Bruni C, Raja J, Denton CP, Matucci-Cerinic M (2015). The clinical relevance of sexual dysfunction in systemic sclerosis. Autoimmun Rev.

[41] Fernandez-Martinez G, Zamora-Legoff V, Hernandez Molina G. Oral health-related quality of life in primary Sjogren’s syndrome. Reumatol Clin. 2018. 10.1016/j.reuma.2018.04.001. 10.1016/j.reuma.2018.04.001 29754950

[42] Liu Z, Dong Z, Liang X, Liu J, Xuan L, Wang J, Zhang G, Hao W (2017). Health-related quality of life and psychological status of women with primary Sjogren’s syndrome: a cross-sectional study of 304 Chinese patients. Medicine (Baltimore).

[43] Mouthon L, Mestre-Stanislas C, Berezne A, Rannou F, Guilpain P, Revel M, Pagnoux C, Guillevin L, Fermanian J, Poiraudeau S (2010). Impact of digital ulcers on disability and health-related quality of life in systemic sclerosis. Ann Rheum Dis.

[44] Peytrignet S, Denton CP, Lunt M, Hesselstrand R, Mouthon L, Silman A, Pan X, Brown E, Czirjak L, Distler JHW (2018). Disability, fatigue, pain and their associates in early diffuse cutaneous systemic sclerosis: the European Scleroderma Observational Study. Rheumatology (Oxford).

[45] Omair MA, Lee P (2012). Effect of gastrointestinal manifestations on quality of life in 87 consecutive patients with systemic sclerosis. J Rheumatol.

[46] Bodukam V, Hays RD, Maranian P, Furst DE, Seibold JR, Impens A, Mayes MD, Clements PJ, Khanna D (2011). Association of gastrointestinal involvement and depressive symptoms in patients with systemic sclerosis. Rheumatology (Oxford).

[47] Maddali-Bongi S, Del Rosso A, Mikhaylova S, Francini B, Branchi A, Baccini M, Matucci-Cerinic M (2014). Impact of hand and face disabilities on global disability and quality of life in systemic sclerosis patients. Clin Exp Rheumatol.

[48] Jaeger VK, Distler O, Maurer B, Czirjak L, Lorand V, Valentini G, Vettori S, Del Galdo F, Abignano G, Denton C (2018). Functional disability and its predictors in systemic sclerosis: a study from the DeSScipher project within the EUSTAR group. Rheumatology (Oxford).

