# Supplementary material for: Health-related quality of life in systemic sclerosis compared with other rheumatic diseases: a cross-sectional study
Source: Arthritis Res Ther. 2019 Feb 15;21:61. doi: 10.1186/s13075-019-1842-x (PMC6377714; doi:10.1186/s13075-019-1842-x)
Supplement: Supplementary file 1 — Table S1. Clinical features of patients with SSc (n=120). (DOCX 16 kb) [file 13075_2019_1842_MOESM1_ESM.docx]

**Supplementary table 1. Clinical features of patients with SSc (n=120).**

|  | n=120 |
| --- | --- |
| Subtype, n (%) | 10.9 (8.3) |
| Diffuse cutaneous SSc | 79 (65.8) |
| Limited cutaneous SSc | 41 (34.2) |
| Modified Rodnan skin score, mean (SD) | 15.6 (10.4) |
| Raynaud’s phenomenon, n (%) | 110 (91.7) |
| Sclerodactyly, n (%) | 82 (68.3) |
| Interstitial lung disease, n (%) | 69 (57.5) |
| Dyspnea, n (%) | 63 (52.5) |
| Reflux symptom, n (%) | 60 (50.0) |
| Telangiectasia, n (%) | 59 (49.2) |
| Arthritis, n (%) | 46 (38.3) |
| Dysphagia, n (%) | 38 (31.7) |
| Digital ulcer, n (%) | 16 (13.3) |
| Pulmonary arterial hypertension, n (%) | 13 (10.8) |
| Calcinosis, n (%) | 10 (8.3) |
