# Supplementary material for: Health-related quality of life in systemic sclerosis compared with other rheumatic diseases: a cross-sectional study
Source: Arthritis Res Ther. 2019 Feb 15;21:61. doi: 10.1186/s13075-019-1842-x (PMC6377714; doi:10.1186/s13075-019-1842-x)
Supplement: Supplementary file 2 — Table S2. SF-36 domains, SF-6D, and EQ-5D-3L scores adjusted by age and sex. (DOCX 19 kb) [file 13075_2019_1842_MOESM2_ESM.docx]

**Supplementary table 2. SF-36 domains, SF-6D, and EQ-5D-3L scores adjusted by age and sex.**

|  | **Group** | | | | | | **Post hoc *p*-value** | | | | | |
| --- | --- | --- | --- | --- | --- | --- | --- | --- | --- | --- | --- | --- |
|  | **SSc**  **(n=120)** | **RA**  **(n=120)** | **SLE**  **(n=120)** | **SjS**  **(n=120)** | **Control (n=600)** | ***P*** | **RA**  **vs.**  **SLE** | **RA**  **vs.**  **SjS** | **RA**  **vs.**  **Control** | **SLE**  **vs.**  **SjS** | **SLE**  **vs.**  **Control** | **SjS**  **vs.**  **Control** |
| PF | 70.5 (2.2) | 70.3 (2.2) | 68.2 (2.2) | 73.9 (2.2) | 82.4 (1.0) | **<0.001** | 0.503 | 0.240 | **<0.001** | 0.071 | **<0.001** | **<0.001** |
| RP | 67.8 (2.3) | 70.5 (2.3) | 65.8 (2.3) | 68.6 (2.4) | 86.5 (1.1) | **<0.001** | 0.151 | 0.547 | **<0.001** | 0.395 | **<0.001** | **<0.001** |
| BP | 61.1 (2.2) | 61.4 (2.2) | 66.1 (2.2) | 63.1 (2.3) | 81.4 (1.0) | **<0.001** | 0.140 | 0.565 | **<0.001** | 0.360 | **<0.001** | **<0.001** |
| GH | 41.4 (1.8) | 51.3 (1.8) | 44.2 (1.8) | 45.8 (1.8) | 63.9 (0.8) | **<0.001** | 0.005 | 0.024 | **<0.001** | 0.540 | **<0.001** | **<0.001** |
| VT | 53.2 (1.9) | 51.8 (1.9) | 49.7 (1.9) | 45.8 (1.9) | 64.6 (0.9) | **<0.001** | 0.427 | 0.022 | **<0.001** | 0.153 | **<0.001** | **<0.001** |
| SF | 72.4 (2.1) | 77.8 (2.1) | 74.6 (2.1) | 71.3 (2.1) | 87.3 (1.0) | **<0.001** | 0.284 | 0.023 | **<0.001** | 0.256 | **<0.001** | **<0.001** |
| RE | 68.4 (2.3) | 77.2 (2.3) | 72.7 (2.3) | 69.6 (2.4) | 87.7 (1.1) | **<0.001** | 0.168 | 0.018 | **<0.001** | 0.357 | **<0.001** | **<0.001** |
| MH | 61.3 (1.8) | 71.7 (1.8) | 67.2 (1.8) | 63.7 (1.8) | 75.2 (0.8) | **<0.001** | 0.077 | **0.001** | 0.086 | 0.172 | **<0.001** | **<0.001** |
| PCS | 44.6 (0.8) | 45.3 (0.8) | 44.8 (0.8) | 46.9 (0.8) | 51.0 (0.4) | **<0.001** | 0.676 | 0.113 | **<0.001** | 0.051 | **<0.001** | **<0.001** |
| MCS | 43.0 (0.9) | 48.9 (0.9) | 46.6 (0.9) | 44.2 (0.9) | 50.8 (0.4) | **<0.001** | 0.074 | **<0.001** | 0.084 | 0.077 | **<0.001** | **<0.001** |
| SF-6D | 0.70 (0.01) | 0.73 (0.01) | 0.72 (0.01) | 0.71 (0.01) | 0.79 (0.00) | **<0.001** | 0.709 | 0.139 | **<0.001** | 0.614 | **<0.001** | **<0.001** |
| EQ-5D-3L | 0.75 (0.01) | 0.81 (0.01) | 0.82 (0.01) | 0.83 (0.01) | 0.90 (0.01) | **<0.001** | 0.065 | 0.295 | **<0.001** | 0.713 | **<0.001** | **<0.001** |

SSc, systemic sclerosis; RA, rheumatoid arthritis; SLE, systemic lupus erythematosus; SjS, Sjogren’s syndrome; PF, physical function; RP, role–physical; BP, bodily pain; GH, general health perception; VT, vitality; SF, social function; RE, role–emotional; MH, mental health; PCS, physical component score; MCS, mental component score; SF-36, Short Form (36) health survey; SF-6D, Short Form Six-Dimensional health index; EQ-5D-3L, three-level version of EuroQol Five-Dimensional descriptive system.
