# Supplementary material for: Health-related quality of life in systemic sclerosis compared with other rheumatic diseases: a cross-sectional study
Source: Arthritis Res Ther. 2019 Feb 15;21:61. doi: 10.1186/s13075-019-1842-x (PMC6377714; doi:10.1186/s13075-019-1842-x)
Supplement: Supplementary file 3 — Table S3. SF-36 domains, SF-6D, and EQ-5D-3L scores adjusted by age, sex, disease duration, comorbidities, and disease activity states. (DOCX 18 kb) [file 13075_2019_1842_MOESM3_ESM.docx]

**Supplementary table 3. SF-36 domains, SF-6D, and EQ-5D-3L scores adjusted by age, sex, disease duration, comorbidities, and disease activity states.**

|  | **Group** | | | | | | **Post hoc *p*-value** | | |
| --- | --- | --- | --- | --- | --- | --- | --- | --- | --- |
|  | | **SSc**  **(n=120)** | **RA**  **(n=120)** | **SLE**  **(n=120)** | **SjS**  **(n=120)** | ***P*** | **RA**  **vs.**  **SLE** | **RA**  **vs.**  **SjS** | **SLE**  **vs.**  **SjS** |
| Physical function | | 66.2 (2.3) | 67.6 (2.3) | 67.3 (2.6) | 61.3 (2.4) | 0.245 | 0.941 | 0.065 | 0.116 |
| Role–physical | | 64.7 (2.5) | 70.3 (2.6) | 66.7 (2.9) | 58.0 (2.7) | **0.011** | 0.378 | **0.001** | 0.037 |
| Bodily pain | | 56.3 (2.3) | 59.7 (2.3) | 69.6 (2.6) | 51.9 (2.4) | **<0.001** | 0.007 | 0.022 | **<0.001** |
| General health | | 37.1 (1.8) | 48.1 (1.8) | 46.8 (2.1) | 38.3 (1.9) | **<0.001** | 0.640 | **<0.001** | 0.005 |
| Vitality | | 48.7 (2.0) | 48.8 (2.1) | 50.4 (2.3) | 38.3 (2.1) | **<0.001** | 0.618 | **<0.001** | **<0.001** |
| Social function | | 70.1 (2.5) | 77.1 (2.5) | 76.0 (2.8) | 64.2 (2.6) | **0.002** | 0.775 | **<0.001** | 0.004 |
| Role–emotional | | 66.4 (2.7) | 76.8 (2.7) | 74.4 (3.0) | 60.7 (2.8) | **<0.001** | 0.563 | **<0.001** | **0.002** |
| Mental health | | 58.3 (2.0) | 69.8 (2.0) | 68.6 (2.3) | 58.2 (2.1) | **<0.001** | 0.701 | **<0.001** | **0.002** |
| Physical component score | | 42.8 (0.8) | 44.3 (0.8) | 45.3 (0.9) | 42.3 (0.8) | 0.068 | 0.414 | 0.081 | 0.020 |
| Mental component score | | 41.8 (1.1) | 48.3 (1.1) | 47.4 (1.2) | 41.5 (1.1) | **<0.001** | 0.612 | **<0.001** | **0.001** |
| SF-6D | | 0.68 (0.01) | 0.72 (0.01) | 0.72 (0.01) | 0.66 (0.01) | **<0.001** | 0.661 | **<0.001** | **<0.001** |
| EQ-5D-3L | | 0.74 (0.02) | 0.81 (0.02) | 0.83 (0.02) | 0.76 (0.02) | **<0.001** | 0.470 | 0.029 | 0.009 |

SSc, systemic sclerosis; RA, rheumatoid arthritis; SLE, systemic lupus erythematosus; SjS, Sjogren’s syndrome; SF-36, Short Form (36) health survey; SF-6D, Short Form Six-Dimensional health index; EQ-5D-3L, three-level version of EuroQol Five-Dimensional descriptive system.
